# O2-6 Older adults' use of neighbourhood open spaces following an environmental intervention

**DOI:** 10.1093/eurpub/ckac094.014

**Published:** 2022-08-29

**Authors:** Tanja Schmidt, Charlotte Skau Palwoski, Jasper Schipperijn

**Affiliations:** Department of Sports Sciences and Clinical Biomechanics, University of Southern Denmark, Odense, Denmark

**Keywords:** Older adults, Social interaction, healthy ageing, Neighbourhood open spaces, Mixed methods

## Abstract

**Background:**

Older age is often associated with functional, social, and mental health problems, leading to major financial burdens on the health care sector, as the number of older adults (65+ years) is expected to increase to 22% by the year 2050. Using neighbourhood open spaces (NOS) for physical activity and social interaction may be an easy way for older adults to uphold healthy and active ageing. However, little is known about older adults' use of NOS. The purpose of this study was to assess factors promoting or inhibiting older adults' use of NOS in a deprived neighbourhood before and after an environmental intervention creating or improving facilities for older adults within NOS.

**Methods:**

A convergent mixed methods approach was used to assess outcomes of a participatory research intervention. Observations using the System for Observing Play and Recreation in Communities were conducted to observe older adults living in a deprived neighbourhood, and their use of 13 NOS in spring 2017 (baseline) and spring 2018 (follow-up). Semi-structures interviews with ten older adults were held in spring 2018 to identify barriers and facilitators for using NOS.

**Results:**

The intervention resulted in the construction of two pavilions and renovated benches, including raised flower beds and small bench-tables. An increase of 44% more older adults was observed at follow-up using the NOS with the renovated benches. No use of the two pavilions was observed. The interviews identified six important factors for older adults' use of NOS: weather, support for social caretakers, support for resourceful volunteers, organized activities, social interaction, and sense of ownership.

**Conclusions:**

Social interaction is a key factor for older adults' use of NOS and should be prioritized by health promoters in combination with age-friendly facilities and organizational support. Daily social interaction may be easier to promote in close distance NOS, whereas NOS further away may rely on organizational support, such as social caretakers and volunteers.

